# Sentinel lymph node mapping for endometrial cancer: Opportunity for medical waste reform☆

**DOI:** 10.1016/j.ygyno.2022.05.008

**Published:** 2022-05-18

**Authors:** Leah A. Marsh, Emeline M. Aviki, Jason D. Wright, Ling Chen, Nadeem Abu-Rustum, Ritu Salani

**Affiliations:** a Department of Obstetrics and Gynecology, Division of Gynecologic Oncology, University of California Los Angeles, Los Angeles, CA, United States of America; b Gynecology Service, Department of Surgery, Memorial Sloan Kettering Cancer Center, NY, NY, United States of America.; c Department of Obstetrics and Gynecology, College of Physicians and Surgeons, Columbia University, New York, NY, United States of America

**Keywords:** Indocyanine green (ICG), Medical waste, Sentinel lymph node dissection, Endometrial cancer

## Abstract

**Objective.:**

As healthcare expenditures continue to rise, identifying mechanisms to reduce unnecessary costs is critical. The objective of this study is to estimate the annual cost of wasted indocyanine green (ICG) used for sentinel lymph node mapping in patients with endometrial cancer.

**Methods.:**

Using the Surveillance, Epidemiology, and End Results program database and Premier database, we determined the annual number of cases in which sentinel lymph node mapping with ICG would be used and the median cost of ICG to institutions and patients, respectively. We assumed that gynecologic oncologists use 2–4 mL (20–40%) of the currently available ICG vial kit (25 mg per 10 mL) per case. Estimated waste was then calculated using cost as a measure of institutional waste and charge as excess cost transferred to patients or payers.

**Results.:**

An estimated 45,864 cases of localized endometrial cancer were identified and eligible for sentinel lymph node (SLN) mapping. The mean total cost associated with ICG was 99.20 and the mean charge was $483.64. The estimated excess annual cost to hospitals was $2,729,825 to $3,639,767. Similarly, using mean charge data, the annual cost of wasted drug for patients and payers was $13,308,999 to $17,745,332.

**Conclusions.:**

The annual cost of wasted ICG due to its current manufactured vial size exceeds $2 million for hospitals and $13.3–$17.7 million for patients. We suggest ICG vials should be packaged in a 10 mg vial kit (2–4 mL sterile solution) to avoid drug waste and the financial impact to institutions and patients.

## 1. Introduction

The advent of novel and personalized pharmaceuticals in oncology has contributed to escalating health care costs and financial hardships to patients. Efforts to reduce these costs include reduction of drug waste. Waste occurs when there is leftover drug in a vial once a patients’ dose has been prepared and the remaining amount cannot be salvaged. This commonly occurs when a drug has variable dosing dictated by bodyweight or body surface area [1].

Cancer drugs covered under Medicare Part B, including intravenous and injectable drugs administered in the hospital or outpatient setting, represent a significant proportion of health care spending [2]. Cancer-related drugs including infusion-based medications are often packaged in quantities larger than the amount needed. Intraoperative agents, like indocyanine green (ICG), are packaged in such a way that only a small amount of the mixture is used. The packaging of both infusion and intraoperative based agents contribute significantly to medical waste.

The ability of a pharmaceutical company to artificially increase the amount of drug they sell per treated patient by increasing the amount in each single dose vial relative to the typically required dose is a known phenomenon. This is further impacted by the limited ability to redistribute any remaining amount of the agent to treat subsequent patients [3]. The financial impact of discarded cancer-related agents is problematic both in Medicare and private insurance markets [4]. In 2019, the Recovering Excessive Funds for Unused and Needless Drug Act or the REFUND Act of 2019 bill was introduced in the Senate. This bill requires drug manufacturers to issue rebates to the Centers for Medicare & Medicaid Services (CMS) in relation to discarded amounts (i.e., amounts remaining after administration) of single-dose vial drugs that are covered under Medicare. Manufacturers that fail to comply are subject to civil penalties (S. 551– 116th Congress 2019–2020) [2].

In early stage disease, endometrial cancer staging surgery has incorporated the use of sentinel lymph node (SLN) dissection [5]. ICG used with fluorescent imaging has emerged as the most consistently effective technique for SLN dissection in endometrial cancer [6]. This technique provides accurate identification of SLN, improves detection of metastatic disease and decreases surgical morbidity [6]. However, the current packaging of ICG is 25 mg/vial and lends itself to a significant portion being wasted. Therefore, we sought to quantify the estimated annual cost of discarded ICG to the healthcare system and the patient in the setting of early-stage endometrial cancer.

## 2. Materials and methods

We used the Surveillance, Epidemiology, and End Results Program (SEER) database to estimate the number of cases of localized endometrial cancer annually. The SEER database is a population-based tumor registry that captures approximately 28% of the U.S. population and contains detailed information on tumor characteristics, sociodemographic features and follow-up [7].

We queried the Premier healthcare database (www.premierinc.com; Premier, Inc., Charlotte, NC) to determine the average cost to institutions and charge to patients associated with ICG. The Premier Healthcare Database is comprised of data from more than 1 billion patient encounters, which equates to approximately one in every five of all inpatient discharges in the U.S. All patient-related data in the database is de-identified and HIPAA compliant from both the inpatient and hospital-based outpatient settings, including demographics, disease state, and information on billed services, including medications, laboratory tests performed, diagnostics and therapeutic services. Premier is de-identified data and was deemed exempt by Columbia University Institutional Review Board. Cost data in Premier include an itemized log of all items that are billed to a patient during hospitalization. Premier reports cost data in the form of billed charges and both fixed and variable costs. For this analysis, we used mean charge and variable cost data associated with use of ICG captured in the database.

We searched Premier for all patients who underwent surgery for endometrial hyperplasia or endometrial cancer from January 2006 to June 2018. Among 142,629 patients, there was a total of 12,543,758 billing records. ICG was identified from the hospital charge description in 7826 records. Therefore, the median charge and cost associated with ICG was estimated from 7826 records. Performance of lymphadenectomy was determined using ICD9 (40.1, 40.11, 40.19, 40.2, 40.29, 40.3, 40.5, 40.50, 40.52, 40.53, 40.59), ICD10 (07TC0ZZ, 07TC4ZZ, 07BC0ZX, 07BC0ZZ, 07BC4ZX, 07BC4ZZ) and CPT (38,500, 38,562, 38,564, 38,570, 38,571, 38,572, 38,589, 38,770, 38,780, 58,960) codes. SLN dissection was defined as either the presence of a CPT for radiopharmaceutical mapping (38,792, 38,900, 78,195, 78,800, 78,801) or of a hospital billing code for ICG, isosulfan blue or technetium-99.

Optimal detection of pelvic SLN occurs when the drug is diluted by the surgeon to a 0.5 mg/mL to 1.25 mg/mL concentration using sterile water [6]. The 25-mg dry powder bottle supplied by the pharmacy is mixed with 10 mL of sterile water in the operating room. Standard practice consists of an ICG injection of 2–4 mL for per case [9].

To perform the drug waste analysis, we assumed that gynecologic oncologists use 2–4 mL of ICG per case. Assuming 10 mL of solution, the presumptive waste per SLN case was 6 mL to 8 mL, or approximately 60–80% of the total vial. Estimated waste was then calculated using mean total cost as a measure of institutional waste and charge as excess cost transferred to patients or payers. The range of hospital cost was calculated by multiplying the number of cases annually by 60% and 80%, which represent the low and high end of wasted drug cost. Sensitivity analyses estimating waste based on only 50% of eligible patients found in SEER was performed. A sensitivity analyses estimating waste based on the 25th and 75th percentile of reported cost and assuming 60% presumptive vial waste was also performed.

## 3. Results

In SEER in 2020, there were 65,520 estimated cases of endometrial cancer [7]. Seventy percent (45,864) of these cases were localized and thus eligible for SLN mapping. In Premier, we identified 142,629 patients who had simple hysterectomy and a diagnosis of uterine cancer (99,860; 70.0%) or endometrial hyperplasia (42,769; 30.0%). Endometrial intraepithelial neoplasia (EIN) was included under endometrial hyperplasia. They had a total of 12,543,758 billing records. Of those, we identified 7826 billing records of ICG. They belonged to 7814 patients, including 7044 (90.2%) with uterine cancer and 770 (9.8%) with endometrial hyperplasia.

Based on the 7826 records evaluated in the Premier database, the mean total cost associated with one vial of ICG was $99.20 (Median: $78.42, Interquartile range (IQR): $49.77–$100.84, Standard deviation (SD): 583.76). The mean charge associated with one vial was $483.64 (Median: $419.34, IQR: $219.79–$527.47, SD: 1674) (Table 1). The mean variable cost associated with one vial of ICG was $67.47 (Median $56.70, IQR: $22.32–$71.00, SD: 472.76).

The total estimated cost of ICG to hospitals associated with current use was then calculated by multiplying the estimated number of endometrial cancer cases (45,864) by the mean total cost ($99.20) which equals $4,556,653. Since 60% to 80% of the vial is wasted depending on whether 2 mL or 4 mL were used, the excess nationwide cost to hospitals accounting for this waste is $2,729,825 to $3,639,767. Similarly, using mean charge data, the annual cost of wasted drug for patients and payers ranges $13,308,999 to $17,745,332 (Table 2).

A sensitivity analysis assuming 50% of eligible patients received SLN biopsy was performed and resulted in an estimated excess nationwide cost to hospitals of $1,364,913 to $1,819,884 and to patients and payers of $6,654,499 to $8,872,666. An additional sensitivity analysis calculating waste based on 25th and 75th percentile of the per-patient cost assuming 60% was wasted resulted in estimated excess cost to hospitals of $1,369,591 to $2,774,955 and estimated cost to patients and payers of $6,048,269 to $14,515,130.

### 3.1. Principle findings

Endometrial cancer is the most common gynecologic cancer in the US with a national estimated expenditure of $5.8 billion dollars in 2020 [10]. In early stage disease, endometrial cancer staging surgery has incorporated the use of SLN dissection with ICG fluorescent imaging. With this, it is important to identify the medical waste associated with this practice. In this setting, our study estimated the annual cost of wasted ICG used for SLN dissection to be $2.7–3.6 million for hospitals and $13.3–17.7 million for patients. This is a gross underestimate as it does not account for other clinical scenarios such as endometrial intraepithelial neoplasia, cervical or vulvar cancer in which SLN dissection could be utilized.

### 3.2. Results in the context of what is known

Medication waste associated with single-use vial drugs is encountered frequently in oncology. Prior studies have evaluated the impact of medication waste associated with systemic cancer-directed therapy. For example, Hatswell and colleagues noted that changing the available vial sizes for drugs may improve usage. Examining pembrolizumab (currently available as 50 mg/100 mg vials), they found that increasing the vial sizes to 70 mg/100 mg could cut drug waste from 13.3% to 8.7% [1]. As Green et al. point out, Part B drugs are priced per unit therefore when vial sizes are limited or larger than necessary, drug is commonly wasted [2]. This practice generates additional profits for the pharmaceutical manufactures, but at the expense of the health care system.

### 3.3. Clinical and research implications

With the current manufacturing of ICG (25 mg per vial) and the typical usage by gynecologic oncologists in early stage endometrial cancer staging, 60–80% of the drug is wasted contributing to millions of dollars of unnecessary costs to hospitals, healthcare payers, and patients. In addition to SLN assessment, FDA indicated uses of ICG are currently limited to hepatic, cardiac and ophthalmic assessments, and ICG is also used in the assessment of bowel anastomosis integrity. Even in these circumstances, it appears a significant portion of the product is wasted [11].

One potential mitigation strategy is to promote vial sharing to allow for more product use from each vial. However, this strategy is limited by regulatory oversight due to concerns with sterility and, even when vial sharing is possible, the potential scheduling and administrative overhead to coordinate patient care will offset some of the savings [1]. Another strategy would be to encourage manufacturers to offer multiple vial sizes which would allow for different price points and reduce waste. It is important to acknowledge that the difference in cost for manufacturers between vial sizes is likely small; however, the way drugs are currently reimbursed is tied to the units of drug offered based on Medicare standards [3]. Therefore, vials that are oversized or one size only result in significant overspending. Further evaluation of the differences in cost of production are important to evaluate and quantify to advocate for this change [3].

### 3.4. Strengths and limitations

The limitations of this study include that it is retrospective in nature. By using the estimated number of patients with localized cancer in the SEER database we are possibly overestimating the cases of patients with endometrial cancer who undergo SLN biopsy with ICG. However, a sensitivity analysis was also performed estimating waste using 50% of eligible SEER patients and still resulted in a waste in excess of $1 million to hospitals and $6 million in excess to patients and payers. Another limitation we would like to acknowledge is the assumption that the relative cost for using a fraction of the entire vial would be a linear reduction versus an exact proportion of the whole. We are unable determine this and assumed all costs were attributed to the cost of the ICG drug itself. We want to recognize the limitations of cost data captured in an administrative data set. Theoretically there should not be considerable different in costs associated with ICG across institutions, yet in this dataset, there were. To account for this, we performed a sensitivity analysis calculating waste based on the 25th and 75th percentile of reported charge and cost data which continued to demonstrate a high amount of waste based on current vial size offered by the manufacturer. Lastly, the calculated waste estimate did not account for cases of endometrial intraepithelial neoplasia which, in many settings, are also managed with SLN biopsy. Future studies evaluating the use of ICG associated with precancerous conditions of the uterus will reveal the magnitude of this medical waste.

## 4. Conclusions

In this manuscript, we estimated the national cost of ICG and applied it to the estimated number of cases of endometrial cancer in US in 2020. The annual cost of wasted ICG due to its current manufactured vial size exceeds $2 million for hospitals and $13.3–$17.7 million for patients. This study sheds light on the extent of drug waste associated with this commonly used intraoperative agent by elucidating the estimated annual excess cost to hospitals and patients. To our knowledge, this is the first study to look at the intraoperative use of ICG associated waste. To address this issue, we recommend that ICG vials should also be available in a 10 mg vial size. While this waste is only a fraction of the total annual cost of endometrial cancer in the United States, identifying areas of waste will slow the rising cost of cancer care, particularly as we incorporate effective and novel, but expensive, therapies for our patients. We anticipate that continuing to identify sources of medical waste, such as dosing of other anti-cancer therapies, and advocating for change will contribute to significant savings in the health care system.

## Figures and Tables

**Table 1:** Mean costs and charge estimates for a 10 cc vial of ICG.

	Mean $	25%ile $	75%ile $	IQR	SD
ICG 10 cc Vial Cost	99.20	49.77	100.84	$49.77 - $100.84	583.76
ICG 10 cc Vial Charge	483.64	219.79	527.47	219.79–527.47	1674

**Table 2:** Total estimated excess cost to Hospitals and Patients from wasted ICG.

	4 mL vial used	2 mL vial used
Estimated annual excess cost to Hospitals	$2,729,825	$3,639,767
Estimated annual excess charge to patients	$13,308,999	$17,745,332

## HIGHLIGHTS

- This study investigated the medical waste associated with SLN dissection in endometrial cancer staging.
- The annual cost of wasted ICG amounts to $2 million to hospitals and $13–17 million to patients.
- Profound medical waste associated with a common practice can be mitigated by modifying ICG manufacturing and packaging.
